# SATB1 in cancer progression and metastasis: mechanisms and therapeutic potential

**DOI:** 10.3389/fonc.2025.1535929

**Published:** 2025-02-25

**Authors:** Jinping Bai, Gege Yang, Qi Yu, Qianya Chi, Xianlu Zeng, Wenjing Qi

**Affiliations:** ^1^ Department of Bioscience, Changchun Normal University, Changchun, China; ^2^ Key Laboratory of Molecular Epigenetics of Ministry of Education, College of Life Sciences, Northeast Normal University, Changchun, China

**Keywords:** SATB1, cancer metastasis, epithelial-mesenchymal transition (EMT), chromatin remodeling, therapeutic strategies

## Abstract

Cancer remains a major global health challenge, with prostate cancer, lung cancer, colorectal cancer, and breast cancer accounting for nearly half of all diagnoses. Despite advancements in cancer treatment, metastasis to distant organs continues to be the leading cause of cancer-related mortality. The progression of cancer involves the alteration of numerous genes, with dynamic changes in chromatin organization and histone modifications playing a critical role in regulating cancer-associated genes. Special AT-rich sequence-binding protein 1 (SATB1), a critical chromatin organizer, plays a pivotal role in cancer progression by regulating gene expression, chromatin remodeling, and cell signaling pathways. SATB1 binds to AT-rich DNA sequences, acting as a scaffold for chromatin-modifying enzymes and transcription factors, thus coordinating the regulation of extensive gene networks. Its overexpression has been implicated in a wide range of cancers and is associated with poor prognosis, aggressive tumor phenotypes, and enhanced epithelial–mesenchymal transition (EMT). Moreover, SATB1’s activity is modulated by microRNAs (miRNAs) and post-translational modifications, further contributing to its complex regulatory functions. Given its crucial involvement in cancer progression and metastasis, SATB1 has emerged as a promising target for novel therapeutic strategies. This review delves into the molecular mechanisms of SATB1 in cancer and explores potential therapeutic approaches for targeting this key regulator in cancer treatment.

## 1 Introduction

Cancer remains one of the most critical global health concerns. As of 2023, prostate cancer, lung cancer, and colorectal cancer (CRC) account for nearly half (48%) of all cancer diagnoses in men, with prostate cancer alone constituting 29% of cases. In women, breast cancer, lung cancer, and CRC represent 52% of new diagnoses, with breast cancer making up 31% of cases (1). Despite declining cancer mortality rates due to advances in medical care, the incidence of certain cancers, such as breast cancer, prostate cancer, and uterine cancer, continues to rise. Most cancer-related deaths result not from primary tumors but from metastasis, where cancer cells spread to distant organs such as the brain, liver, lungs, and bones (2). Although the mechanisms driving cancer metastasis are not fully understood, epithelial–mesenchymal transition (EMT) has been recognized as a critical factor (3). EMT is a process where epithelium-derived tumors acquire mesenchymal characteristics, leading to loss of cell polarity and intercellular adhesion, coupled with cytoskeletal reorganization (3–7). A hallmark of EMT is the loss of E-cadherin (CDH1) expression, a protein essential for cell–cell adhesion and maintaining cellular polarity (8–10). While the progressive loss of genetic stability and accumulation of mutations are thought to deregulate EMT-related genes in malignant cells (11), some evidence suggests that cancer cells may acquire metastatic potential before full malignancy, indicating that EMT may occur early in cancer progression (12). Consequently, cancer cells may gain the ability to metastasize soon after the initial oncogenic mutation, allowing them to migrate and colonize distant organs.

The accumulation of genetic mutations is a key driver of cancer development, with as few as three mutations sufficient to initiate tumorigenesis (13). After initiation, cancer progresses through additional mutations, accompanied by abnormal gene expression patterns. Various genes are altered during cancer progression, and factors such as epigenetic modifications, gene fusions, and chromosomal translocations add layers of complexity to the molecular landscape of cancer (14). Chromatin structure and organization are fundamental in gene regulation, as epigenetic events can activate or silence gene transcription (15). Studies have shown that dynamic changes in chromatin organization and histone modifications are essential for regulating cancer-associated genes, thereby promoting tumor growth and survival by driving proliferation, differentiation, and evasion of apoptosis (15–17). Significant changes in gene regulation often necessitate alterations in chromatin structure, which involves recruiting chromatin remodeling enzymes and epigenome-modifying enzymes to specific genomic regions, alongside the assembly of transcription factors. Special AT-rich sequence-binding protein 1 (SATB1), a chromatin organizer, plays a critical role in reprogramming the cell’s gene expression profile, enabling rapid phenotypic shifts (18). SATB1 regulates gene networks across the genome, controlling 2–10% of the human genome (19–22). Aberrant SATB1 expression is associated with various cancers, including breast cancer, lung cancer, and CRC. In most cases, high SATB1 expression correlates with an aggressive phenotype and poor prognosis (23–27). Moreover, SATB1 has been implicated in promoting EMT, a critical process in tumor metastasis (25, 28–30). The newly generated mesenchymal-like cells in EMT express biomarkers such as N-cadherin, Vimentin and, β-catenin, which indicate tumor progression and invasiveness. Several transcriptional regulators, including Snail, Slug and ZEB1, induce EMT by downregulating epithelial markers and upregulating mesenchymal markers (7). SATB1 modulates the expression of these genes at the transcriptional level, inhibiting CDH1 expression and promoting the conversion of cancer cells to a mesenchymal phenotype (18, 25, 26, 31–34). Moreover, SATB1 regulates EMT through key signaling pathways, including Wnt/β-catenin and Notch (22, 32, 35–40). Regarding the oncogenic function of Wnt/β-catenin, its upregulation occurs in human cancers and it can accelerate EMT-mediated metastasis and drug resistance (41). SATB1 regulates the activation and stabilization of β-catenin, a central mediator of the Wnt signaling pathway (22, 32, 39, 40). When SATB1 is present, it enhances the accumulation of β-catenin in the nucleus, where it interacts with T-cell factor/lymphoid enhancer-binding factor (TCF/LEF) transcription factors to activate the expression of EMT-related genes (35, 39). In addition, overexpression of SATB1 was shown to significantly promote the expression of the Notch receptors Notch1 and Notch4, as well as the downstream target of Notch signaling, Hes-1 (37). The activation of Notch signaling pathway by SATB1 drives the expression of Snail1 and Twist1 genes in EMT (37). Furthermore, it was found that miR-448 suppression leads to enhanced SATB1 expression, which initiates amphiregulin—epidermal growth factor receptor signaling towards Twist1 expression through mitogen activated protein kinase and nuclear factor κB (NF-κB) activation (42, 43). In this review, we will explore the molecular mechanisms underlying SATB1’s contribution to cancer progression and metastasis, as well as the potential strategies for targeting SATB1 in cancer treatment.

## 2 Overview of SATB1

**Figure 1:**
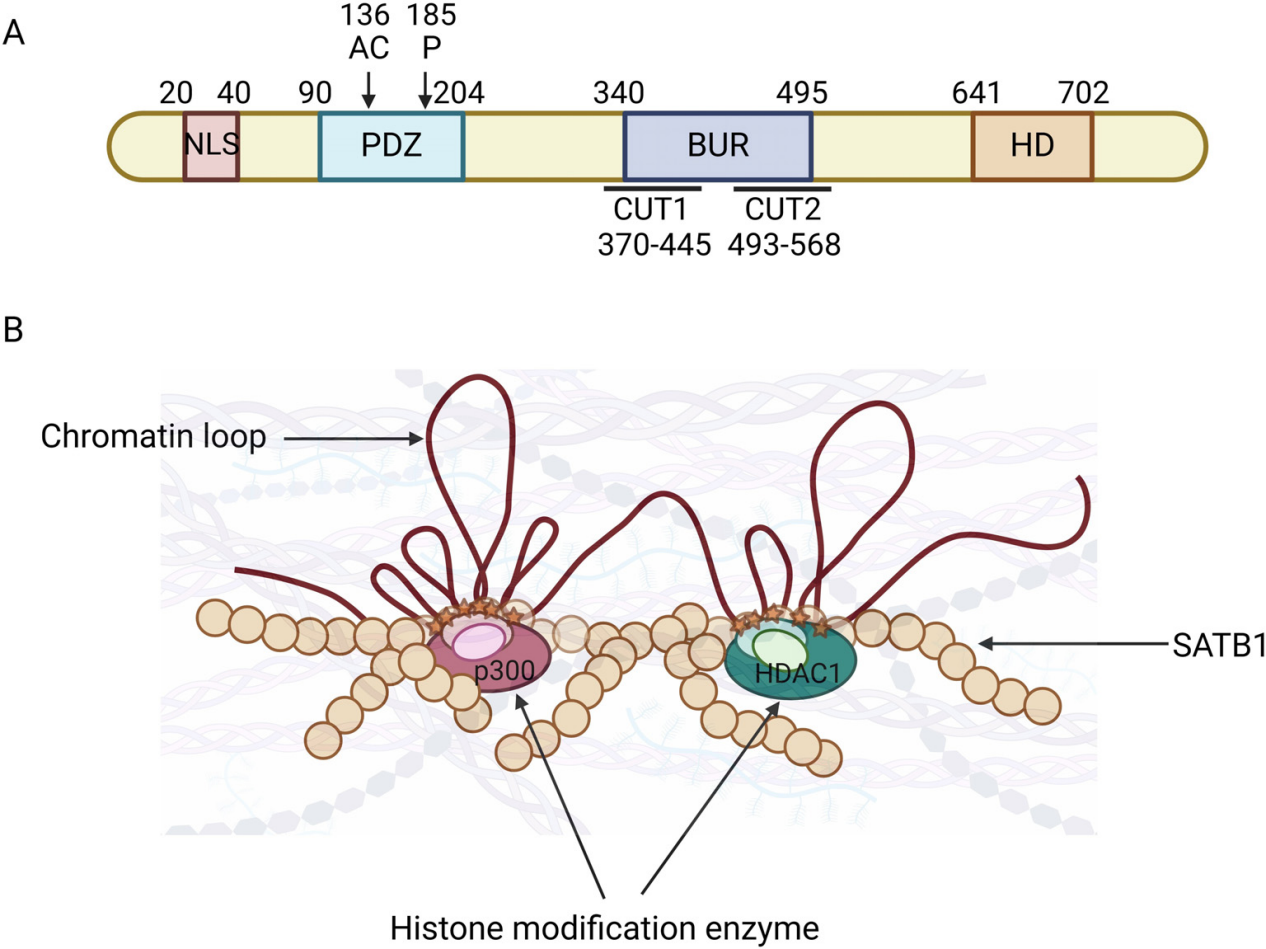
Domains and function of special AT-rich sequence-binding protein 1 (SATB1). **(A)** Schematic representation of SATB1 protein. SATB1 is a nuclear matrix protein whose primary protein structure and functional domains include the nuclear localization signaling domain (NLS), the protein binding domain (PDZ), the base unpairing regions (BURs), and the DNA-binding motif homeodomain (HD). The BUR and HD domains confer DNA-binding specificity and affinity, while the PDZ domain facilitates interactions with chromatin-modifying enzymes, making SATB1 a versatile gene activator or repressor. [Figure adapted from http://atlasgeneticsoncology.org/Genes/SATB1ID44225ch3p24.html]. **(B)** SATB1 mediates chromatin loop formation. SATB1 (yellow circle) binds to the BUR sequence (red star) within specific target genes, anchors to the nuclear matrix to form a chromatin ring, mediates long-distance gene regulation, and provides docking sites for histone modification enzymes (such as histone acetylase p300 and histone deacetylase 1 (HDAC1)) and transcription factors. Phosphorylation of SATB1 strengthens its interaction with HDAC1 and increases DNA-binding affinity. Acetylation of SATB1 leads to its dissociation from DNA, release HDAC1, and activate gene expression. [Figure adapted from (18, 50)].

Initially identified in 1992 by Dickinson et al., SATB1 is a nuclear-matrix-associated protein that binds to AT-rich DNA sequences, forming a scaffold for chromatin-modifying enzymes and transcription factors (19, 20, 44). Genomic regions characterized by the ATC sequence context are called base-unpairing regions (BURs). Most genes have several major BURs within 100 kb 5’, in introns, and 100 kb 3’ (18). Chromatin is organized into a non-random three-dimensional topology, and within this framework, SATB1 modulates the chromatin structure around the CCCTC-binding factor, a global chromatin remodeler that marks the boundaries of many topologically associating domains (18, 45, 46). SATB1 also binds to the genomic regions outside heterochromatin, precisely at the base of chromatin loop domains suggesting its role in the positioning of genes in regions of the nucleus where their expression can be modulated (20). Furthermore, SATB1 is postulated to enhance transcription by promoting the formation of small chromatin loops locally between regulatory elements, which would reduce the physical volume of within the gene cluster (43). Chromatin looping is not only important for chromatin compaction, but it is thought to be involved in gene regulation—a distal regulatory sequence could be brought to close proximity with a locus, or multiple co-inducible genes could be brought together to be co-regulated (18). SATB1 not only folds chromatin into loops via binding to BURs but also provides a nuclear platform to recruit chromatin remodeling and modifying enzymes to loci around the BURs (18). It recruits histone deacetylases and other histone-modifying enzymes to these regions, resulting to histone modifications that either activate oncogenes or repress anti-oncogene (18, 47–49) (**Figure 1**). SATB1 is predominantly expressed in T-cell lines, including thymocytes and TH2 cells, and plays an essential role in T-cell development, homeostasis, early erythroid differentiation, and responses to physiological stimuli (18, 23, 51). SATB1 is indispensable for T lymphocyte development (21, 48) through its involvement in properly organizing nuclear architecture, especially chromatin folding (18, 51). It has been shown to regulate TH2 cell differentiation through long-range chromosomal interactions (51, 52) and is functionally linked to the Wnt/β-catenin signaling pathway (35). By recruiting β-catenin, SATB1 influences TH2 cell differentiation in a Wnt-dependent manner (53), and both Wnt/β-catenin and SATB1 share several key target genes, such as c-Myc and Bcl-2 (21, 54, 55). Cancer typically originates from genetic mutations and chromosomal instability, with these changes often closely associated with alterations in chromatin. Structural changes in chromatin may directly contribute to the initiation and proliferation of cancer cells. Frequent and preferential juxtaposition of gene loci, such as *Myc* and *Igh*, in the nucleus of B lymphocytes has been observed, predisposing cells to chromosomal translocations (56, 57). Several studies have showed that SATB1 affects *Myc* and *Igh* gene expression, thereby affecting chromatin stability and cancer cell progression (55, 58, 59). SATB1’s ability to regulate chromatin organization and gene expression underscores its role in cancer biology, particularly in metastasis.

Structurally, SATB1 comprises six functional domains: the nuclear localization signaling (NLS) domain; the protein binding domain (PDZ) domain, facilitating protein–protein interactions; the BUR-binding domain, including the CUT1 and partial CUT2 domains; and the homeodomain (HD), a DNA-binding motif (50) (**Figure 1**). The CUT1 domain is critical for efficient chromatin binding, contributing significantly to SATB1’s high-affinity binding. The HD domain, on the other hand, ensures binding specificity, interacting with DNA features like negative propeller Twist and high AT contents (60, 61). The BUR and HD domains confer DNA-binding specificity and affinity, while the PDZ domain facilitates interactions with chromatin-modifying enzymes, making SATB1 a versatile gene activator or repressor. These functions are further regulated by post-translational modifications including acetylation and phosphorylation (62–64). Phosphorylation of SATB1 acts as a molecular switch regulating its transcriptional activity (62). For instance, phosphorylation via protein kinase C at serine 185 enhances SATB1’s interaction with histone deacetylase 1 (HDAC1), increasing its DNA-binding affinity and repressive activity (62). By contrast, SATB1 dephosphorylation leads to acetylation via P300/CBP-associated factor at lysine 136, causing SATB1 to dissociate from DNA, release HDAC1, and derepress or activate gene expression (63). SIRT1 has also been reported to deacetylate SATB1 (65). It has been shown that dynamic modulation of SATB1 acetylation status determines its oncogenic potential (66). HDAC5 binds to and deacetylates SATB1 at the conserved lysine 411 residue, and the dynamic regulation of acetylation at this site is determined by TIP60 acetyltransferase (66). HDAC5-mediated deacetylation promotes SATB1-dependent repression of tumor suppressor genes, and deacetylated SATB1 also represses SDHA-induced epigenetic remodeling and anti-proliferative transcriptional program (66). SATB1 regulates gene expression by mediating histone modifications as it recruits histone-modifying enzymes on promoters of oncogenes and also recruits HDAC1 on promoters of tumor suppressor genes (19, 23). SATB1 also undergoes ubiquitination, with UBE3A, an E3 ubiquitin ligase, mediating its degradation. MiR-218-5p targeted and negatively regulated UBE3A expression to inhibit ubiquitin-mediated SATB1 degradation (67). SMURF2, another E3 ubiquitin ligase, promotes SATB1 degradation by upregulating its ubiquitination, and its deficiency promotes cancer cell proliferation and SATB1 target gene transcription (68). Additionally, ubiquitin-specific peptidase 47 (USP47), a member of the deubiquitinating enzymes family, interacts with SATB1 and mediates its deubiquitination and stability (68, 69). USP47 deficiency impairs transcriptional activity of SATB1 target genes and inhibits proliferation, migration, and tumorigenesis (68, 69). These findings suggest that the post-translational modifications of SATB1 is critical for its function.

SATB1 expression is also regulated by multiple microRNAs (miRNAs), including miR-1224, miR-409, miR-448, miR-21, miR-155, miR-302a-3p, miR-21-5p, and miR-495-3p (42, 70–77). miRNAs are small, non-coding RNAs (20–22 nucleotides) involved in post-transcriptional regulation, and their role in modulating oncogenes is critical in cancer invasion and metastasis (78–80). For instance, elevated miR-191 levels in epidermal keratinocytes induce cellular senescence by downregulating SATB1 and cyclin-dependent kinase 6 (81), while in breast cancer, miR-191 promotes tumorigenesis by downregulating SATB1 (82). Conversely, miR-23a acts as a tumor suppressor in osteosarcoma by inhibiting SATB1 expression, reducing SATB1 mRNA and protein levels and suppressing cell proliferation (83). SATB1 is also regulated by miR-21, a well-known oncogenic miRNA implicated in various cancers, including glioblastoma, lung cancer, gastric cancer, breast cancer, liver cancer, cervical cancer, and ovarian cancer (70–77, 82, 84–86). These findings highlight SATB1 as a target of multiple miRNAs, further modulated by post-translational modifications and tissue-specific localization. Additionally, tumor-derived transforming growth factor β decreased SATB1 expression through promoting the binding of Smad proteins to the *Satb1* promoter (87, 88). Similarly, NF-κB signaling and IL-4 signaling regulate SATB1 expression via alternative promoter usage during Th2 differentiation (89). Recent studies showed that SATB1 regulates key genes involved in carcinogenesis, including ERBB2, KAI1, KISS1, and CDH1 (18). Its expression is positively correlated with several biological and genetic markers, including cyclin D1, matrix metalloproteinase-2 (MMP-2), NF-κB, and proliferating cell nuclear antigen, while negatively correlated with APC and BRAF expression (38). SATB1 overexpression can transform non-invasive cells into invasive, tumorigenic cells (23), while SATB1 knockdown in highly invasive cancer cells restores normal morphology and reduces their migratory and invasive capabilities (23, 38, 90) (**Table 1**). Given its central role in regulating multiple genes and pathways, SATB1 represents a promising target for tumor-specific therapies.

**Table 1:** Special AT-rich sequence-binding protein 1 (SATB1) expression and cancers.

Cancer type		SATB1 expression and function	References
Reproductive and Urinary system tumor	Breast cancer	• SATB1 expression increases and correlates with tumor differentiation.	(23, 37, 47, 71, 91–94)
		• Stimulates the epithelial–mesenchymal transition (EMT)-related transcription factors Snail and Twist1, and promotes metastasis.	
		• Upregulates HER2 and negatively correlates with TLR4; regulated by miR-409, H19, and miR130a-3p.	
		• A factor in poor prognosis and metastasis.	
	Ovarian cancer	• SATB1 expression increases significantly.	(24, 29, 95)
		• Reprograms ovarian cancer’s energy metabolism by regulating LDH and MCT1, thus promoting cancer metastasis.	
		• A factor in poor prognosis and metastasis.	
	Endometrial cancer	• SATB1 expression increases.	(96–99)
	Prostate cancer	• SATB1 expression significantly increases and correlates with cell invasion and migration.	(26, 28, 100–104)
		• Downregulates E-cadherin (CDH1), stimulates MMP-9 expression, and induces invasive tumors by promoting EMT.	
	Bladder cancer (BLCA)	• SATB1 is upregulated and promotes metastatic features.	(31, 105, 106)
Respiratory system tumor	Lung cancer	• SATB1 expression level varies in different lung cancer subtypes.	(30, 36, 70, 90, 107–109)
		• Correlates with key EMT-related proteins: CDH1, N-cadherin, Snail, Slug, and Twist1. • Affected by post-translational modification by acetylation of HDAC5	
		• Loss of expression leads to poor prognosis in squamous cell carcinomas (SCC).	
		• Upregulated in small cell lung cancer (SCLC) and associated with proliferation and invasion.	
Alimentary system tumor	Colorectal cancer (CRC)	• SATB1 expression increases and correlates with tumor progression and metastasis.	(32, 33, 35, 39, 110–116)
		• Both a Wnt/β-catenin pathway target and a regulator of β-catenin expression in CRC. • Influences the various transfer-related proteins, including CDH1, N-cadherin, Slug, Twist1, and MMP7.	
		• Indicates poor prognosis.	
	Gastric cancer	• SATB1 expression increases significantly; associated with a significant reduction in survival.	(34, 77, 117–119)
		• Positively correlates with N-cadherin and Vimentin and negatively correlates with CDH1.	
		• High SATB1 expression with reduced chemotherapy agent sensitivity; promotes chemotherapy resistance in gastric cancer.	
	Esophageal squamous cell carcinoma (ESCC)	• SATB1 expression increases and negatively correlates with survival time.	(120–122)
		• Upregulates fibronectin 1 (FN1) and platelet-derived growth factor receptor beta (PDGFRB) in esophageal cancer.	
	Pancreatic cancer	• SATB1 is overexpressed and promotes the proliferation and invasion of cancer cells, and its expression level is closely related to the depth and stage of tumor invasion.	(58, 123, 124)
		• Increases chemotherapy sensitivity but associated with lower survival rate.	
	Liver cancer	• Associated with the development, recurrence, and metastasis of liver cancer. • Changes the gene expression profile of live cancer.	(25, 125–128)

## 3 SATB1 in reproductive and urinary system tumors

### 3.1 Breast cancer

breast cancer is one of the most prevalent malignancies in women, and its incidence is increasing significantly, particularly among younger populations (129). It is estimated that approximately one in eight women in developed countries will be diagnosed with breast cancer (130). In its early stages, breast cancer is often asymptomatic, leading to delayed diagnoses. Approximately 15% of patients are diagnosed with metastatic disease at initial presentation, which complicates timely treatment (131). Despite significant advances in therapeutic interventions, effectively managing metastatic breast cancer continues to be a challenge.

SATB1 has been identified as a key regulator in breast cancer progression and metastasis through its role in reprogramming gene expression networks (18, 23). The seminal study by Han et al. in 2008 laid the foundation for understanding SATB1’s involvement in breast cancer, showing that SATB1 mRNA and protein are predominantly expressed in metastatic breast cancer cell lines compared with non-malignant breast tissues. High levels of SATB1 expression were found to correlate with poor tumor differentiation and advanced disease stages (23). Zhang et al. further confirmed this finding, showing that SATB1 was abundantly expressed in breast cancer specimens but almost undetectable in normal and non-malignant tissues. Additionally, SATB1 expression gradually increased as breast tissues progressed from cystic hyperplasia to precancerous lesions, eventually reaching advanced breast cancer stages (91). Meanwhile, Kobierzycki et al. also reported a positive association between SATB1 expression and breast cancer progression, although their findings lacked statistical significance (132). These findings highlight its potential role in early cancer development.

**Figure 2:**
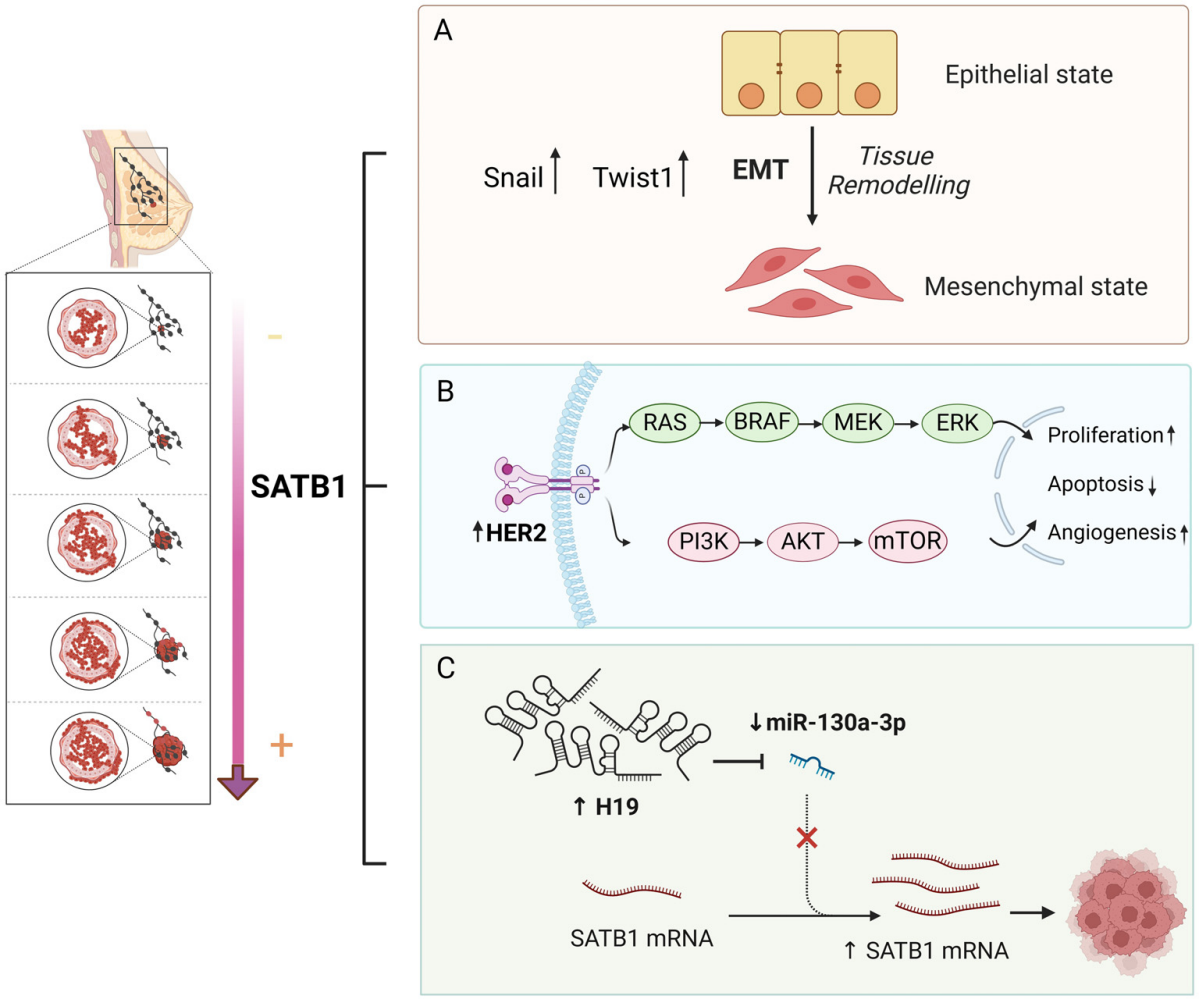
Molecular mechanism of SATB1 in breast cancer. **(A)** SATB1 stimulates the expression of the most critical EMT-related transcription factors, Snail and Twist1, providing breast cancer cells with a strong distant metastatic ability. **(B)** SATB1 plays a synergistic role with HER2 in breast cancer. SATB1 can directly upregulate HER2 expression, promote cell proliferation, inhibit cell apoptosis and promote angiogenesis through a series of signaling pathways. **(C)** MiR130a-3p downregulates SATB1. H19, a long non-coding RNA, sponges miRNA-130a-3p, resulting in SATB1 upregulation, thus promoting breast cancer progression.

Han et al. also reported that SATB1 promotes metastatic potential and invasiveness in breast cancer. They found that the ectopic expression of SATB1 in non-metastatic cells, inducing invasive behaviors and SATB1 knockdown in metastatic breast cancer cells (MDA-MB-231), reversed their invasive phenotype and prevented both lung metastases and primary tumor formation in mice (23). This gene expression analysis revealed that SATB1 modulates around 1,000 genes related to cell adhesion, signaling, and metastasis (23). However, a subsequent study by Iorns et al. using similar cell lines reported that SATB1 knockdown did not significantly affect invasiveness, and its overexpression failed to promote metastasis in non-invasive cells (133). Additionally, their clinical analysis showed no correlation between SATB1 expression and overall survival (OS) in patients with primary breast cancer (133). This inconsistency could be due to differences in cell line heterogeneity and RNA probe specificity (134). Further studies have reinforced the association between SATB1 and breast cancer aggressiveness. Wang et al. observed that SATB1 expression negatively correlated with TLR4 expression, which positively correlated with tumor size, stage, local lymph node metastasis, and estrogen receptor (ER) protein levels (135). Sun et al. found that SATB1 increased the breast cancer stem cell population within tumors and enhanced the expression of key EMT-related transcription factors, such as Snail and Twist1, further driving breast cancer metastasis (37). Additionally, Ma et al. revealed that the flavonoid baicalein effectively reduced breast cancer metastasis by inhibiting both SATB1 and the Wnt/β-catenin signaling pathway (22). This finding was further corroborated by Gao et al., who showed that baicalein suppressed breast cancer cell proliferation and migration by downregulating SATB1 (136). Notably, SATB1 was also found to act synergistically with HER2, an oncogene pivotal in breast cancer progression. SATB1 directly upregulated HER2 expression, thereby enhancing the tumorigenic potential of breast cancer cells (23, 92, 137). SATB1 is also regulated by non-coding RNAs, including miRNAs and long non-coding RNAs. Chen et al. identified SATB1 as a downstream effector gene of miR-409, revealing a negative correlation between the expression levels of these two genes. miR-409 was found to regulate the biological behavior of breast cancer cells by targeting SATB1. Furthermore, altering SATB1 levels could mitigate the effects of miR-409 on breast cancer cell proliferation and invasion (71). Additionally, SATB1 expression has been shown to positively correlate with H19, a long non-coding RNA that plays a crucial role in tumorigenesis (93, 138, 139). H19 sponges miRNA-130a-3p, resulting in SATB1 upregulation, thus promoting breast cancer progression (93) (**Figure 2**). These studies highlight the multifaceted role of SATB1 in promoting breast cancer progression and metastasis.

The prognostic significance of SATB1 in breast cancer has been debated. Han et al. initially reported that high SATB1 expression was linked to poorer OS in patients with breast cancer (23). However, Hanker et al. observed no significant relationship between SATB1 expression and OS in ER-negative breast cancer, though it emerged as a positive prognostic marker in ER-positive cases (94). By contrast, Liu et al. identified SATB1 as an independent negative prognostic marker, with SATB1 positivity correlating with a significantly higher risk of poor outcomes (92). Laurinavicius et al. confirmed SATB1’s prognostic value, showing that a high Ki67/SATB1 ratio predicted worse OS in hormone-receptor-positive breast cancer (140). Nonetheless, conflicting results have emerged from other studies. For instance, Iorns et al. and Patani et al. found no significant association between SATB1 expression and patient outcomes (133, 141). Despite these conflicting results, a trend linking higher SATB1 expression to more aggressive disease and reduced OS persists. A 2016 meta-analysis by Pan et al. further validated SATB1’s role in breast cancer progression, associating its high expression with an advanced tumor stage, lymph node metastasis, and decreased survival rates (47). In summary, while inconsistencies exist in the literature, most evidence points to SATB1 as a key player in breast cancer progression and metastasis. Its potential role as a prognostic marker, particularly in predicting aggressive disease, warrants further investigation.

### 3.2 Ovarian cancer and uterine cancer

Ovarian cancer remains a leading cause of mortality among gynecological malignancies, primarily because of its metastatic potential and high recurrence rates. SATB1 overexpression has been observed in epithelial ovarian cancer, where it is positively associated with FIGO stages, lymph node metastasis, and poor prognosis (24, 29). SATB1 knockdown in ovarian cancer cells reduces lactate dehydrogenase (LDH) and monocarboxylate transporter 1 (MCT1) expression, key mediators of glucose metabolism. Simultaneously, tumor suppressor genes such as BRCA1 and BRCA2 are upregulated (95). These findings suggest that SATB1 may promote ovarian cancer metastasis by modulating cellular energy metabolism.

In lung cancer, particularly endometrial cancer, SATB1 expression is significantly higher in cancerous tissues than in non-cancerous tissues (96–98). It plays a critical role in promoting invasion and metastasis, in part through interactions with PAK5, a kinase involved in EMT regulation (98, 99). The PAK5-SATB1 axis may also play a critical role in the progression of other cancers. SATB1 expression in lung cancer correlates with tumor grade, infiltration depth, and lymph node metastasis (96, 97). Additionally, SATB1 has been identified as an independent negative prognostic marker in patients with endometrial cancer (97). In cervical cancer, high SATB1 expression is associated with poorer survival outcomes (142). While SATB1’s exact mechanisms in ovarian cancer and lung cancer progression remain under investigation, its role as a negative prognostic factor is well established.

### 3.3 Prostate cancer

prostate cancer is the second most common cause of cancer-related mortality among men worldwide (143). SATB1 has been identified as a key player in prostate cancer metastasis. Shukla et al. demonstrated that SATB1 expression significantly increases with advancing tumor grade (26). Mao et al. confirmed these findings, showing that SATB1 was expressed in prostate cancer tissues but was absent in benign prostatic hyperplasia. Moreover, SATB1 expression was positively correlated with the Gleason score, though it did not correlate with patient age or prostate-specific antigen levels, indicating its role in tumor progression rather than early detection (100).

Further research supports the critical role of SATB1 in maintaining the invasive potential of prostate cancer cells. Increased SATB1 expression has been positively correlated with enhanced cell invasiveness and migration (26, 100). In prostate cancer cell lines (PC-3M, DU-145, and LNCaP), SATB1 knockdown markedly reduces cell proliferation, invasion, and growth (26, 28, 100, 144). Moreover, SATB1 downregulation elevates CDH1 levels and reduces MMP-9 expression, reversing the EMT process and thereby restoring a more differentiated, less invasive phenotype (26). SATB1 knockdown in DU-145 cells also reduces cell viability, adhesion, and invasive capacity (28, 101–103). Conversely, SATB1 overexpression in LNCaP cells promotes xenograft tumor growth and induces EMT-related protein expression, further enhancing prostate cancer cell proliferation, invasion, and migration (102). Similarly, the transient transfection of SATB1 in PZ-HPV-7 cells increases their migratory and invasive capacities by 50–67%, along with downregulating CDH1 and upregulating MMP-9 (26). An innovative approach developed by Mao et al. utilized a lysosomal adenovirus vector carrying SATB1 shRNA (ZD55-SATB1), which selectively targets and replicates in DU-145 and LNCaP cells. This method significantly downregulated SATB1 expression, effectively reducing cell viability and invasion *in vitro* and *in vivo* (144). Overall, SATB1 consistently emerges as a promoter of proliferation, invasion, and migration in prostate cancer, while its downregulation triggers nuclear consolidation and apoptosis, particularly in DU-145 cells (102, 103). These findings highlight SATB1’s critical role in driving prostate cancer progression and its potential as a therapeutic target.

### 3.4 Bladder cancer

BLCA is ranked as the sixth most common cancer in men worldwide (143), and it has also been linked to SATB1 overexpression. SATB1 is highly expressed in both BLCA cell lines and clinical samples, with elevated levels correlating with shorter survival times for patients with BLCA (31, 105, 106). Loss- and gain-of-function studies have revealed that SATB1 plays a pivotal role in BLCA cell proliferation, migration, apoptosis, and sensitivity to cisplatin-based chemotherapy (31, 106). Notably, SATB1 promotes EMT in BLCA by downregulating CDH1 and upregulating key EMT-related transcription factors such as Snail, Slug, and Vimentin. This EMT activation enhances the invasive and metastatic capacities of BLCA cells. There is a strong inverse correlation between SATB1 and CDH1 expression at both the mRNA and protein levels, reinforcing SATB1’s role in promoting EMT and metastasis (31). Furthermore, SATB1 expression is positively correlated with critical clinical parameters, such as primary tumor invasion depth, lymph node metastasis, and TNM stages (31, 105). These associations underscore SATB1’s clinical relevance as a potential biomarker for aggressive disease and poor prognosis in patients with BLCA.

## 4 SATB1 in respiratory system tumors

lung cancer remains one of the most prevalent malignancies worldwide, consistently ranking as the leading cause of cancer-related deaths (145). lung cancers are broadly categorized into two primary subtypes: small-cell lung carcinoma (SCLC), accounting for approximately 20% of cases, and non-small-cell lung carcinoma (NSCLC), representing about 80% of cases. NSCLC can be further classified into adenocarcinoma (AC) and squamous cell carcinoma (SCC) (107, 146). SATB1 plays a crucial role in lung cancer progression, with varying impacts depending on the histological subtype.

### 4.1 Non-small-cell lung carcinoma

Research on SATB1 in lung cancer has primarily focused on NSCLC. Selinger et al. observed that, although SATB1 expression was significantly lower in NSCLC samples than in normal bronchial tissues, its expression varied by histological subtype. SATB1 expression was notably higher in SCC than in AC, where elevated levels were associated with poor differentiation and early-stage disease (108). In contrast, some studies have reported elevated SATB1 expression in AC tissues compared with adjacent non-malignant lung tissues (70, 90). These discrepancies likely reflect distinct SATB1 expression patterns across different lung tissues. SATB1 is generally highly expressed in the normal bronchial epithelium but nearly absent in alveolar cells (107). In NSCLC, SATB1 expression in AC is correlated with tumor hypo-differentiation, while in SCC, higher levels of SATB1 are found in well-differentiated tumors. Additionally, SATB1 expression has been positively associated with the proliferation marker Ki67 in SCC, though this correlation does not extend to AC (107). In the aggressive AC cell line A549, SATB1 knockdown significantly inhibits cell proliferation, migration, and invasion but promotes apoptosis, underscoring SATB1’s role in fostering a more aggressive tumor phenotype (90).

In 2021, Glatzel-Plucinska et al. explored the relationship between SATB1 expression and key EMT-related proteins in NSCLC clinical samples, observing strong positive correlations with EMT markers. Specifically, SATB1 showed a significant correlation with SLUG in SCC and Twist1 in AC, indicating its role in driving tumor progression via EMT in both subtypes (30, 90, 108). Further experiments revealed that SATB1 expression increased following EMT induction in NSCLC cell lines, such as A549 and NCI-H1703, supporting SATB1’s role as a positive regulator of EMT (32). Additionally, SATB1 knockdown in NSCLC cells downregulates tumor-promoting genes like c-myc, MMP2, MMP9, S100A4, and VEGF-B while also reducing the expression of EMT-related genes (e.g., N-cadherin, fibronectin) and inhibiting the Wnt/β-catenin pathway. Meanwhile, genes promoting apoptosis, such as Bcl-2, IL-2, and IL-2R, are upregulated (32, 36). By studying H1299, H358 and BEAS-2B cells and biochemical screening, Shalakha et al. determined that SATB1 is a novel HDAC5 substrate. HDAC5 deacetylates SATB1 at the conserved Lys 411 site. The dynamic acetylation at this site is determined by TIP60 acetyltrasferase. Significantly, HDAC5-mediated deacetylation is critical for SATB1-dependent downregulation of key tumor suppressor genes. HDAC5-SATB1 axis modulates the cellular transcriptional profile to promote tumor development (66). These findings indicate that SATB1 drives tumor growth and metastasis through multiple pathways, including the Wnt/β-catenin pathway, and its post-translational modifications could also be important to understand its role in tumorigenesis.

The prognostic role of SATB1 in NSCLC remains debated. Selinger et al. identified SATB1 deletion as a poor prognostic factor in SCC, but not in AC (108). Lower SATB1 levels have also been linked to shorter OS in patients with NSCLC and a history of smoking (108). However, Glatzel-Plucinska et al. reported that elevated SATB1 levels were a positive prognostic factor in NSCLC overall (AC and SCC combined), though the results were only marginally significant (107). These conflicting findings highlight the complexity of SATB1’s role in lung cancer and underscore the need for further research.

### 4.2 Small-cell lung carcinoma

SATB1’s role in SCLC is less understood than that in NSCLC. SCLC is an aggressive cancer characterized by rapid growth and early metastasis. Despite its initial responsiveness to chemotherapy and radiotherapy, SCLC remains challenging to treat owing to frequent relapses (146). Selinger et al. reported higher SATB1 levels in SCLC samples compared with NSCLC, suggesting a different role for SATB1 in these subtypes (108). Interestingly, elevated SATB1 expression in SCLC was linked to better prognosis, although the small sample size limits the generalizability of these findings (108). Huang et al. further demonstrated higher SATB1 expression in SCLC tissues and metastatic lymph nodes than in adjacent normal tissues, reinforcing the idea that SATB1 contributes to SCLC progression (109). In the NCI-H446 SCLC cell line, SATB1 knockdown significantly inhibited proliferation and invasion while promoting apoptosis (109). In summary, SATB1 plays a multifaceted role in lung cancer, with its effects varying according to histological subtype. In NSCLC, SATB1 is implicated in EMT and tumor progression, while its role in SCLC, though less clear, also appears to promote aggressive cancer behavior. Although some findings suggest SATB1’s potential as a prognostic marker, further research is necessary to fully understand its clinical implications and therapeutic potential in both NSCLC and SCLC.

## 5 SATB1 in alimentary system tumors

### 5.1 Colorectal cancer

Despite advances in diagnostic and therapeutic strategies, the prognosis for advanced CRC remains poor, primarily because of its high metastatic potential (143). In 2011, Meng et al. demonstrated that SATB1 protein and mRNA were significantly overexpressed in CRC tissues, especially in patients with early-onset rectal cancer. Increased SATB1 levels correlated with higher metastatic potential and were significantly elevated in high-metastatic cells compared with low-metastatic ones (110). Subsequent studies have consistently confirmed that SATB1 is overexpressed in CRC samples relative to adjacent non-malignant tissues (32, 38, 111–115, 147, 148), with its overexpression associated with deeper tumor infiltration, lymph node metastasis, poor differentiation, and advanced TNM stages (32, 38, 110–112, 114, 115). Further studies also showed that the expression of SATB1 in lymph node metastasis was higher than that in primary lesion, and that in distant organ metastasis was higher than that in primary lesion (39). The increased expression of SATB1 correlates with expression profiles of various epigenetic factors including KDM6A, KDM6B and EMT factors Snail and ZEB1 suggesting a crosstalk between various EMT factors, SATB1 and epigenetic factors which drives the cancer towards metastasis associated (116).

**Figure 3:**
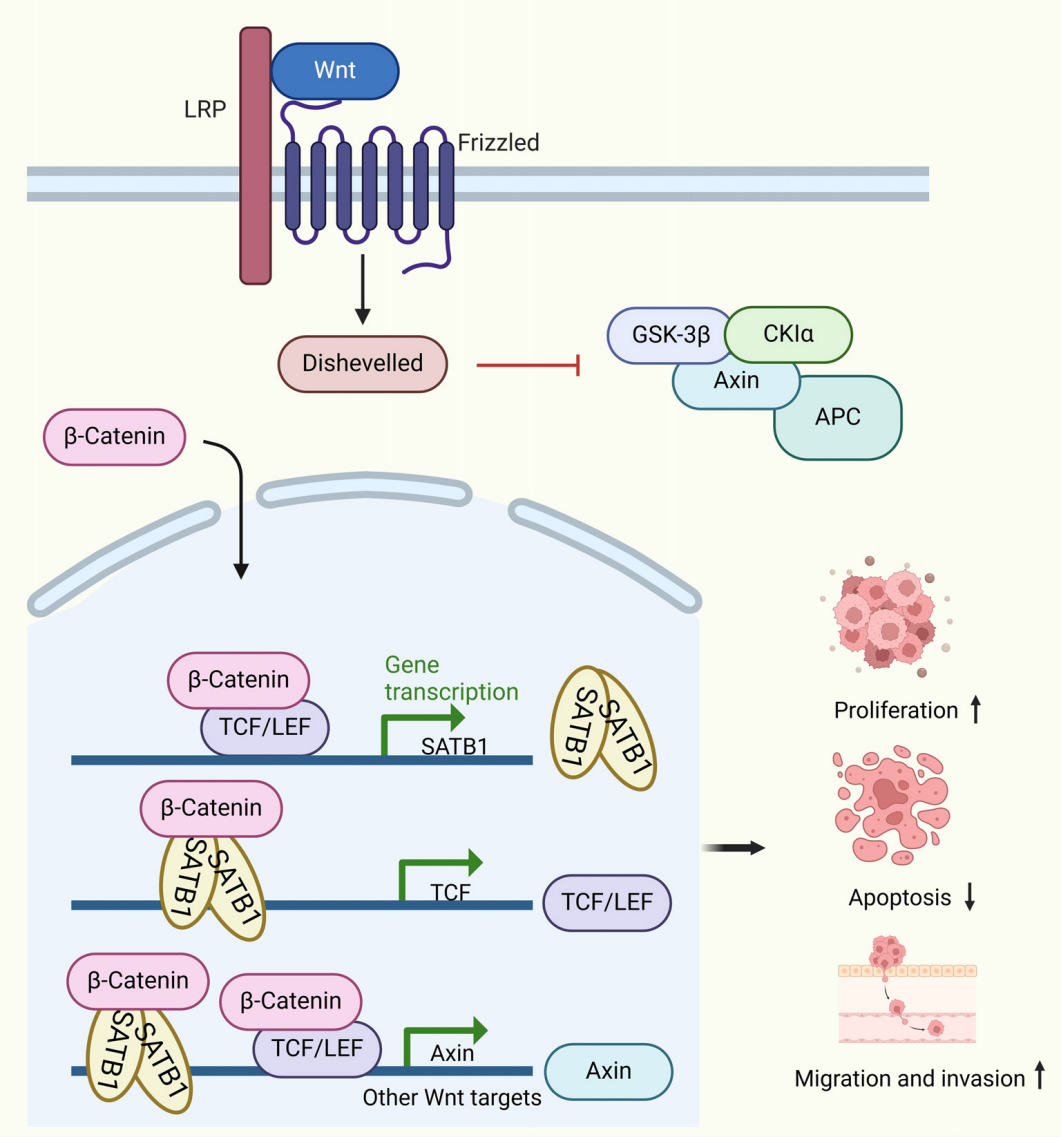
Molecular mechanisms of SATB1 in colorectal cancer (CRC). SATB1 overexpression leads to the nuclear accumulation of β-catenin. Following the nuclear accumulation of β-catenin, the TCF7L2/β-catenin complex binds to the satb1 promoter, inducing its expression. The SATB1/β-catenin complex binds to the TCF7L2 promoter to maintain its expression. The TCF7L2/β-catenin and SATB1/β-catenin complexes then bind to the Wnt response gene to induce its expression. This positive feedback loop leads to cancer progression.

SATB1’s role in CRC progression is partly attributed to its involvement in dysregulating the Wnt/β-catenin signaling pathway, which is critical for EMT in CRC. In normal colorectal mucosa, β-catenin is predominantly localized to cell membranes, but in CRC tissues with high SATB1 expression, β-catenin is found in the cytoplasm and nucleus. Luan et al. further confirmed that SATB1 induced β-catenin translocated into the nuclear and promotes colorectal cancer tumorigenesis by *in vitro* and *in vivo* experiments (39).This shift is associated with reduced membrane-bound β-catenin, increased EMT markers (e.g., Vimentin), and decreased CDH1 and CK20 levels, highlighting SATB1’s role in promoting metastasis (32). Furthermore, SATB1 not only acts as a target of the Wnt/β-catenin pathway but also regulates β-catenin expression (35). Wnt/β-catenin pathway hyperactivation induces SATB1 expression, which is repressed when TCF7L2 (TCF4) and β-catenin are depleted. SATB1, in turn, binds to the *TCF7L2* promoter and modulates downstream Wnt signaling targets (35). Several studies have also shown that SATB1 knockdown in CRC cells reduces growth, migration, invasion, and colony formation while promoting apoptosis (38, 39, 110, 114, 115). SATB1 knockdown affects the expression of metastasis-related proteins, including CDH1, N-cadherin, Slug, Twist1, and MMP7, confirming its role in EMT and extracellular matrix degradation (33) (**Figure 3**). *In vivo* studies further demonstrate that silencing SATB1 in LS174T cells injected into mice can significantly reduce tumor growth or even completely inhibit tumor formation (33). Conversely, SATB1 overexpression accelerates tumor growth and promotes metastasis to the liver and lungs (115).

The prognostic significance of SATB1 in CRC is controversial. While some studies suggest that SATB1 is an independent marker of poor prognosis (111–115), others have found no such association (147) or report prognostic value only in SATB2-negative tumors (24). Some studies even propose that high SATB1 expression may be associated with improved OS (149). Zhao et al. reported that high SATB1 expression is associated with shorter OS, low tumor differentiation, and distant metastasis but found no correlation between SATB1 and TNM stages (150). These findings suggest that further research is needed to clarify the mechanisms through which SATB1 impacts CRC prognosis, potentially related to its expression variants and the stage of cancer development.

### 5.2 Gastric cancer

Gastric cancer remains a leading cause of cancer-related deaths, especially in East Asia and Eastern Europe (143). SATB1 is significantly overexpressed in gastric cancer tissues compared with normal gastric mucosa (77, 117), with high SATB1 expression associated with poor survival, local invasion, lymph node metastasis, and advanced TNM stages (34, 117, 118, 151, 152). SATB1 overexpression is also linked to EMT promotion in gastric cancer, characterized by increased N-cadherin and Vimentin, along with reduced CDH1, correlating with advanced disease and lymph node metastasis (34). Furthermore, SATB1 has been implicated in the regulation of multidrug resistance (MDR) in gastric cancer, contributing to poor chemotherapy responses and worse outcomes (117, 118).

Long non-coding RNA UCA1 (lncRNA-UCA1) has emerged as a promising biomarker for early detection and prognostic prediction in gastric cancer, with elevated levels linked to poorer OS and disease-free survival (153). Additionally, miRNA-495-3p functions as a tumor suppressor in gastric cancer, where its loss leads to oncogene overexpression, promoting malignant transformation and tumor growth, while its overexpression inhibits tumor growth and metastasis (154, 155). Sun et al. explored the relationship between SATB1, miR-495-3p, and lncRNA-UCA1, revealing that SATB1 knockdown in gastric cancer cells inhibited cell proliferation and invasion, induced apoptosis, and mirrored the effects of miR-495-3p overexpression and lncRNA-UCA1 suppression (77). Their findings demonstrated that SATB1’s 3′-untranslated region (3′-UTR) and lncRNA-UCA1 competitively bind to miR-495-3p, with a luciferase reporter assay, confirming that miR-495-3p binds to multiple sites on SATB1’s 3′-UTR and a single site on lncRNA-UCA1 (77). SATB1 knockdown increased miR-495-3p binding to lncRNA-UCA1, reducing lncRNA-UCA1 expression. These results suggest that SATB1 3′-UTR functions as a competing endogenous RNA (ceRNA) for miR-495-3p, positively regulating lncRNA-UCA1. Interestingly, lncRNA-UCA1 knockdown reduced SATB1 expression in MKN-45 cells but not in BGC-823 cells (77), indicating a cell-dependent regulatory mechanism between lncRNA-UCA1 and SATB1 in gastric cancer. Similarly, LINC00491 accelerated head and neck squamous cell carcinoma progression through regulating miR-508-3p/SATB1 axis (156). Also, lncRNA IGF-like family member 2 antisense RNA 1 functioned as a ceRNA to sponge miR-1224-5p to regulate the expression of SATB1, regulating the Wnt/beta-catenin signaling pathway and tongue squamous cell carcinoma progression (157).

MDR remains a major challenge in treating gastric cancer, contributing to high mortality rates. Luo et al. found that high SATB1 expression in gastric cancer is associated with reduced sensitivity to various chemotherapeutic agents, promoting chemoresistance both *in vitro* and *in vivo* (119). SATB1 enhances the activity of ATP-binding cassette (ABC) transporter proteins—key players in reducing drug accumulation in cancer cells, thus promoting MDR—by altering their subcellular localization rather than expression (158). SATB1 also regulates the *Ezrin* promoter, which interacts with ABC transporters, contributing to chemoresistance (119). Receptor tyrosine kinases of the HER family, particularly HER2 and HER3, have been suggested to compensate for reduced MET function, contributing to resistance against MET inhibitors (159). SATB1 is implicated in the upregulation of HER3 expression following MET inhibition, enhancing HRG/HER3 signaling and thereby allowing gastric cancer cells to evade the cytotoxic effects of MET-targeted therapies (160). Additionally, SATB1 expression correlates positively with HER2, indicating a potential regulatory relationship between SATB1 and HER2 (117). Thus, targeting SATB1 offers a promising strategy for overcoming drug resistance in gastric cancer, potentially enhancing the effectiveness of chemotherapy. In conclusion, SATB1 plays a pivotal role in the progression, chemoresistance, and metastasis of gastric cancer, positioning it as a potential therapeutic target for improving treatment outcomes in patients with gastric cancer.

### 5.3 Esophageal cancer

SATB1 has been closely linked to the progression of esophageal cancer, particularly esophageal squamous cell carcinoma (ESCC), the most common form of esophageal cancer, arising primarily from abnormal squamous epithelial cell proliferation (161). Studies have shown that SATB1 expression is significantly higher in ESCC tissues than in normal esophageal tissues, and its expression is strongly associated with TNM stages, though not with other clinicopathologic characteristics (120, 121). Importantly, patients with elevated SATB1 levels have significantly shorter survival times than those with lower SATB1 expression, making SATB1 a predictive marker for recurrence and poor prognosis in ESCC (120, 121).

Gene Ontology and Kyoto Encyclopedia of Genes and Genomes pathway analyses have identified fibronectin 1 (FN1) and platelet-derived growth factor receptor beta (PDGFRB) as key genes regulated by SATB1 in TE-1 cells (122). FN1 and PDGFRB are highly expressed in human esophageal cancer and play significant roles in promoting cell proliferation and migration. In other cancers, FN1 is known to stimulate the expression of matrix metalloproteinases, promoting invasion and metastasis (162), while PDGFRB supports the growth and survival of glioma stem cells (163) and breast cancer cell migration (164). Although the precise roles of these genes in esophageal cancer are not yet fully understood, their involvement in cancer progression is evident. A study by Song et al. revealed that SATB1 knockout in TE-1 and EC-109 cells significantly reduced the mRNA and protein levels of FN1 and PDGFRB (122). Moreover, a luciferase reporter assay confirmed that the activity of the *FN1* and *PDGFRB* promoters increased approximately 2.5-fold following SATB1 transfection (122). These results suggest that SATB1 upregulates FN1 and PDGFRB, contributing to their oncogenic roles in ESCC by promoting cell survival and migration. Thus, SATB1 is a promising therapeutic target and prognostic marker in esophageal cancer.

### 5.4 Pancreatic cancer

Pancreatic cancer is one of the most lethal malignancies globally, with its incidence continuing to rise. SATB1 is overexpressed in pancreatic cancer, playing a critical role in promoting cancer cell proliferation and invasion. SATB1 expression is closely associated with tumor invasion depth and disease stage (58, 123). SATB1 knockdown has been shown to significantly inhibit cell proliferation, colony formation, and non-adherent growth while reducing the migratory capacity of pancreatic cancer cells *in vitro*. *In vivo* studies have also demonstrated that SATB1 downregulation suppresses tumor growth in xenograft models (123, 165). Additionally, elevated SATB1 expression is associated with shorter survival times in patients with pancreatic cancer (123, 124). Mechanistically, SATB1 binds to specific regions of the *myc* promoter, inducing *myc* mRNA expression, in turn promoting cancer cell growth, increasing the number of S-phase cells, and enhancing invasiveness *in vitro*. Conversely, SATB1 knockdown suppresses cancer cell proliferation and invasion (58). Blocking MYC in SATB1-overexpressing cells attenuates these effects, highlighting the SATB1–MYC axis as a potential therapeutic target (58). Recent research has also revealed that deleting deoxynucleotidyltransferase terminal-interacting protein 2 (DNTTIP2) reduces SATB1 expression, which regulates cyclin-dependent kinase 6 (CDK6), and directly controls cyclin-dependent kinase CDK1. This regulatory pathway leads to G1 phase arrest in MIA-PaCa-2 cells and G2 phase arrest in PK-1 cells, thereby inhibiting pancreatic cancer cell proliferation (166). These findings underscore the potential of SATB1 as a therapeutic target in pancreatic cancer.

### 5.5 Liver cancer

Liver cancer is the third leading cause of cancer-related deaths worldwide (143). SATB1 is highly expressed in human hepatocellular carcinoma (HCC) tissues and in HCC cell lines with high metastatic potential, driving tumor growth *in vivo* (25). Clinical studies show that SATB1 expression correlates with larger tumor size, poor differentiation, and lymph node metastasis (25). Similarly, in intrahepatic cholangiocarcinoma, SATB1 expression has been associated with lymph node involvement and distant metastasis (167). *In vitro* studies further demonstrate that high SATB1 levels are associated with an aggressive cellular phenotype (25, 125). Research by Wei Tu et al. highlighted that SATB1 is strongly expressed in HCC cell lines with high metastatic potential, indicating a correlation between SATB1 expression and aggressive tumor behavior (126–128). Furthermore, SATB1 influences gene expression in HCC cells, regulating over 300 genes involved in tumor growth and metastasis (25). *In vivo* studies show that SATB1 promotes cell cycle progression by upregulating cyclin-dependent kinase 4 and downregulating p16INK4A, inhibiting apoptosis through the FADD–caspase-8–caspase-3 pathway. SATB1 also facilitates EMT, as evidenced by increased Snail1, Slug, Twist, and Vimentin expression and decreased CDH1, ZO-1, and desmoplakin expression (25). These findings suggest that SATB1 plays a critical role in HCC development by regulating key genes involved in cell cycle progression, apoptosis, and EMT, making it a potential therapeutic target in liver cancer.

## 6 Strategies for targeting SATB1 in cancer therapy

Cancer initiation, invasion, and metastasis are highly complex processes, involving numerous factors such as key proteases, regulatory proteins, and associated signaling pathways. Targeting and disrupting one or more of these processes is a crucial strategy in cancer therapy. The development of anti-invasive and anti-metastatic drugs with low toxicity and high efficacy is of significant theoretical and clinical importance, as these advancements offer the potential to improve therapeutic outcomes, enhance prognosis, and improve the quality of life of patients with cancer. This section explores chemical drugs and gene therapy strategies that specifically target SATB1 (**Table 2**).

**Table 2:** SATB1-targeting therapeutic strategies for cancer.

Methods		Drugs or Carriers	Source	Cancer treated	Mechanisms
Chemical Drugs		Baicalein	*Scutellaria baicalensis* Georgi	breast cancer, liver cancer, pancreatic cancer (22, 136, 168–170)	Downregulates SATB1 expression, leading to vimentin, and Snail, Wnt1, and β-catenin inhibition and enhanced CDH1 expression in MDA-MB-231 cells.
		Statin drugs (mainly hydrophobic statins such as fluvastatin)	–	breast cancer, ovarian cancer, prostate cancer, colon cancer, non-small-cell lung cancer (NSCLC) (171–176)	Promotes the post-translational modification of SATB1 and induces the proteolytic degradation of SATB1. Fluvastatin downregulates SATB1 through Wnt/β-catenin pathway.
		Triptolide	*Tripterygium wilfordii* in the Euonymus family	CRC, melanoma, breast cancer, ESCC (177–180)	Modulates the circNOX4/miR-153-3p/SATB1 axis.
		Ganodermanontriol	*Ganoderma lucidum*	Liver cancer (181)	Inhibits SATB1 gene expression; regulates Bcl-2, Bax, and caspase 3 expression; and induces cell apoptosis.
		Nicotinamide (Nano-HAPs)	A water-soluble vitamin and component of vitamin B3	Teratocarcinoma (182)	Downregulation of SATB1 expression promotes growth arrest and apoptosis in MF9 cells.
Gene Carrier Therapeutics	Virus carrier	ZD55-SATB1+DTX	–	prostate cancer (144, 183)	Increases caspase-3 and caspase-8 and decreases Bcl-2 and CD31, promoting apoptosis in DU145 and PC-3 cells. This is more effective than monotherapy and is non-cytotoxic to WPMY-1 cells.
	Non-viral carriers (including liposomes and other nanoparticles)	TSMCL-DOX-shSATB1		gastric cancer (184)	DOX and SATB1 shRNA vectors can both be guided to the tumor site under magnetic field guidance, and DOX can be released in a high-temperature-triggered manner to achieve drug therapy.
		CD44-SATB1-ILs		gastric cancer (185)	SATB1 siRNA-encapsulated immune liposomes binding to CD44 antibodies target cancer-initiating cells (CICs), reducing CIC proliferation by about 80% and reducing the CIC population by about 60%.
		Hydroxyapatite nanoparticles (Nano-HAPs)		Human glioma (186–188)	shRNA-SATB1 delivery using Nano-HAPs significantly inhibits the growth, invasion, and angiogenesis of human glioma U251 cells *in vitro* and *in vivo*.

### 6.1 Chemical drugs

#### 6.1.1 Baicalein

**Figure 4:**
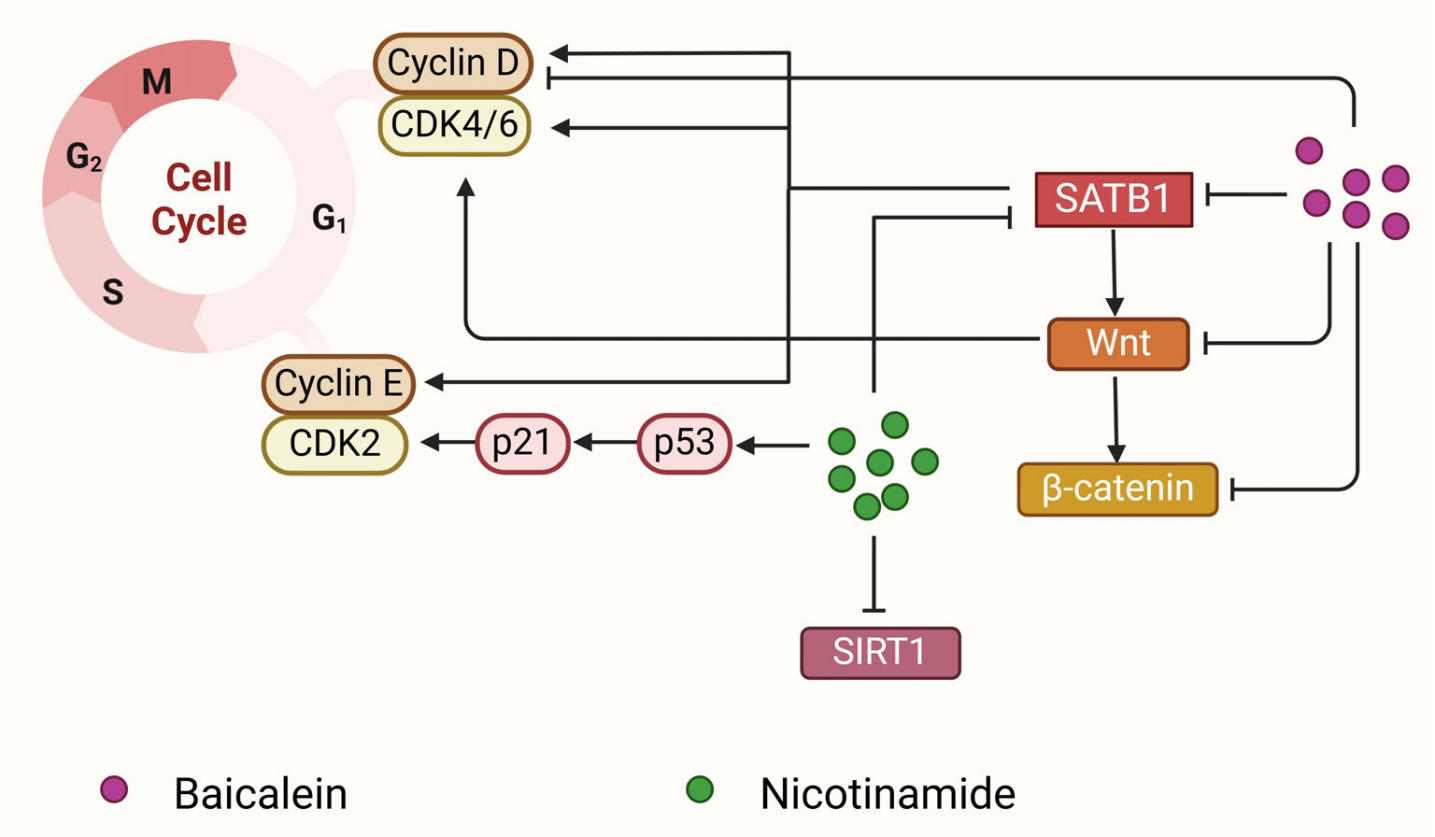
Baicalein and nicotinamide block the cell cycle to inhibit cancer proliferation. Baicalein (purple circle) downregulates SATB1 expression and then inhibits Wnt and β-catenin expression, which further induces cell cycle arrest by decreasing cyclin D1 expression. Nicotinamide (green circle) can decrease SATB1 expression, inhibit SIRT1 expression, and upregulate the p53 acetylation, together inhibiting cyclin-dependent kinase 2 and cyclin-dependent kinase 4/6 and leading to G1 arrest. SATB1 regulates the expression of CDK4/6, cyclin D1 and cyclin E, and promotes cell cycle progression and cell proliferation.

Baicalein (molecular formula: C_15_H_10_O_5_; molecular weight: 270.24 g/mol) is a bioactive flavonoid monomer isolated from *Scutellaria baicalensis* Georgi, the primary active ingredient in Radix Scutellariae (136). Traditionally, *S. baicalensis* has been used to treat inflammation, hypertension, cardiovascular diseases, and bacterial and viral infections, making it one of the most versatile herbal medicines in China (189). Extensive research has confirmed that *S. baicalensis* and its flavonoids possess significant anticancer properties, including apoptosis induction, cell cycle arrest, EMT inhibition, reactive oxygen species elimination, and tumorigenesis prevention (189–192). Baicalein has been reported to suppress the proliferation, invasiveness, and metastatic potential of human breast cancer, liver cancer, and pancreatic cancer cells (168–170). It also enhances the efficacy of chemotherapeutic agents in multidrug-resistant tumor cells (169, 170). In breast cancer, baicalein inhibits the proliferation and metastasis of MCF-7, MDA-MB-231, BT549, and 4T-1 cells by downregulating MMP-2 (136). More importantly, baicalein reduces SATB1 expression, downregulates Vimentin and Snail, and increases CDH1 levels, thereby inhibiting EMT and reducing distant metastasis in breast cancer. Additionally, baicalein suppresses Wnt1 and β-catenin proteins, further contributing to EMT inhibition (22, 136, 193) (**Figure 4**). The antitumor effects of baicalein are mediated through various signaling pathways, including NF-κB, PI3K/Akt, mTOR, and TGF-β/Smad (189, 192, 194, 195), making it a promising therapeutic agent for targeting SATB1 in cancer treatment.

#### 6.1.2 Statin drugs

3-Hydroxy-3-methylglutaryl coenzyme A reductase inhibitors, commonly known as statins, are widely used to treat hypercholesterolemia by reducing serum cholesterol levels (196, 197). Statins have also recently been shown to inhibit tumor growth and metastasis *in vitro* and *in vivo* at clinically relevant doses (171–175). However, the precise molecular mechanisms by which statins induce cancer cell death remain unclear. One study demonstrated that SATB1 is downregulated by statins in a time- and dose-dependent manner in COLO 205 cells (198). This effect appears to be specific to hydrophobic statins, such as simvastatin and fluvastatin, and not to hydrophilic pravastatin (198), consistent with previous findings on the differential activities of statins in breast cancer and gynecological cancers (171, 176). Interestingly, statins reduce SATB1 protein levels without affecting SATB1 transcription, suggesting that statins promote the proteasome-mediated degradation of SATB1. Proteasome inhibitors have been shown to restore SATB1 protein levels in statin-treated cells, indicating post-translational modifications that lead to SATB1 degradation through the proteasome pathway (198). Among the statins, fluvastatin has demonstrated effectiveness in reducing tumor proliferation and promoting apoptosis in various cancers, including breast cancer (172), prostate cancer (173), OC (174), and HCC (175). Its efficacy has been confirmed *in vivo* (199, 200). Additionally, fluvastatin significantly inhibits tumor progression in NSCLC H292 cells, potentially by downregulating SATB1 via the Wnt/β-catenin pathway (36). Further studies are needed to elucidate the mechanisms by which statins regulate SATB1 in other cancers.

#### 6.1.3 Triptolide

**Figure 5:**
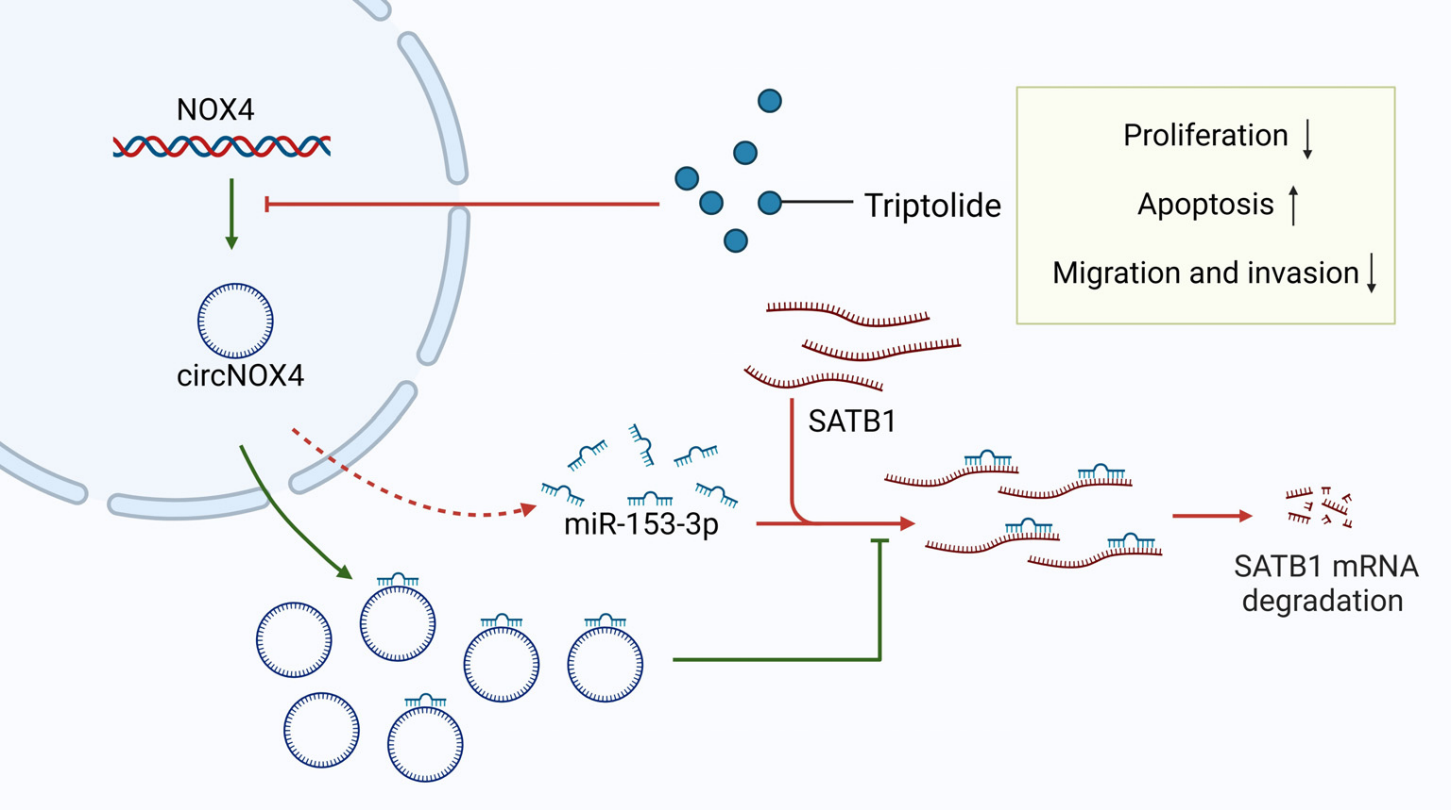
Triptolide inhibits esophageal squamous cell carcinoma (ESCC) proliferation and migration by regulating the circNOX4/miR-153-3p/SATB1 signaling pathway. CircNOX4 and SATB1 are highly expressed in ESCC, while miR-153-3p expression is low. MiR-153-3p acts as a tumor suppressor and downregulates SATB1 by binding to the 3’UTR of SATB1. CircNOX4 positively regulates SATB1 by sponging miR-153-3p. Triptolide downregulates circNOX4 and then releases miR-153-3p, degrading SATB1 and thereby inhibiting cell proliferation and migration.

Triptolide is a biologically active compound derived from *Tripterygium wilfordii* and exhibits a broad spectrum of biological activities. Previous studies have demonstrated that triptolide effectively inhibits the growth of various cancer cell lines, including colon cancer, melanoma, and breast cancer (177–179, 201–203). Recent findings reveal that triptolide inhibits the progression of ESCC, primarily by modulating the circNOX4/miR-153-3p/SATB1 axis (180). Circular RNAs (circRNAs)—non-coding RNAs involved in transcriptional and post-transcriptional regulation (204, 205)—often act as sponges for miRNAs, thereby regulating downstream mRNA expression (206, 207). Abnormal circRNA expression has been linked to cancer development (208, 209). CircNOX4 promotes cancer cell proliferation and metastasis in CRC (210, 211). Similarly, miR-153-3p acts as a tumor suppressor in various cancers, including HCC (212), gastric cancer (213), and oral cancer (214). CircNOX4 positively regulates SATB1 by sponging miR-153-3p, and its expression is elevated in ESCC tissues and cells (180, 215). Triptolide reduces circNOX4 expression, which downregulates SATB1, thereby inhibiting cell proliferation, migration, and EMT (180) (**Figure 5**). miR-153-3p overexpression blocks circNOX4’s effects on triptolide-induced ESCC cell proliferation. Since SATB1 is a target of miR-153-3p, SATB1 knockdown mitigates the pro-proliferative effects of miR-153-3p inhibitors in triptolide-treated ESCC cells (180). These findings suggest that triptolide is a potent therapeutic agent targeting SATB1 in ESCC, though further studies are needed to explore its efficacy in other cancers.

#### 6.1.4 Other chemical drugs

In addition to baicalein, statins, and triptolide, other chemical compounds have demonstrated potential in inhibiting tumorigenesis by regulating SATB1 expression, including ganodermanontriol and nicotinamide. Ganodermanontriol, a triterpenoid isolated from *Ganoderma lucidum*, significantly inhibits HCC cell proliferation and metastasis. It may downregulate SATB1 expression, thereby inducing apoptosis and suppressing tumor growth by regulating apoptosis-related proteins such as Bcl-2, Bax, and caspase-3 (181). Nicotinamide, a water-soluble form of vitamin B3, has been shown to induce apoptosis in F9 mouse teratocarcinoma stem cells (MF9) by downregulating SATB1 expression, thus promoting growth arrest and apoptosis in these cells (182) (**Figure 4**). The ongoing advancement of medical research continues to unveil new drugs targeting SATB1. These therapies aim to precisely target specific genes or proteins in cancer cells, minimizing damage to normal cells, improving treatment outcomes, and reducing side effects.

### 6.2 Gene therapy approaches

Gene therapy has emerged as a promising treatment strategy for various diseases, particularly cancer, by targeting and modifying specific genes within cells. This approach offers significant advantages, including high specificity and minimal side effects, and, in some cases, holds the potential for a curative outcome (216, 217). However, a major challenge in gene therapy lies in the effective delivery of therapeutic genes. Delivery systems are generally categorized into viral and non-viral vectors, with non-viral vectors such as liposomes and nanoparticles gaining favor owing to their low immunogenicity.

#### 6.2.1 Viral vectors

Mao et al. developed a lysogenic adenovirus (ZD55) carrying SATB1 shRNA to investigate its effects on prostate cancer growth and metastasis. Their findings demonstrated potent antitumor activity in human prostate cancer models (144). When ZD55-SATB1 was combined with the chemotherapeutic agent Docetaxel (DTX) in human prostate cancer cell lines (DU145 and PC-3), it significantly inhibited cell proliferation, migration, and invasion while promoting apoptosis more effectively than either treatment alone. Notably, this combination therapy exhibited no cytotoxicity in normal stromal cells (WPMY-1). In animal models, the combination of ZD55-SATB1 and DTX significantly reduced tumor growth, increased pro-apoptotic factors such as caspase-3 and caspase-8, and decreased levels of anti-apoptotic proteins such as Bcl-2, along with reductions in angiogenic markers (CD31) (183). This novel approach shows great promise for treating prostate cancer.

#### 6.2.2 Non-viral vectors

Hydroxyapatite Nanoparticles (Nano-HAPs): Nanomedicine integrates nanotechnology with medical therapeutics and has shown substantial promise in gene therapy. Nanoparticles are used for diagnostics and targeted therapies, offering advantages such as low immunogenicity, repeatable administration, and high specificity in RNA interference (RNAi) delivery. Chu et al. utilized nano-HAPs to deliver SATB1 shRNA, significantly inhibiting growth, invasion, and angiogenesis in human glioma U251 cells both *in vitro* and *in vivo*. Nano-HAPs alone inhibited glioma cell proliferation, but their combination with SATB1 shRNA enhanced these effects, demonstrating the potential of this approach for glioma therapy (186, 187).

Thermosensitive Magnetic Liposomes: Liposomes have been widely used to improve the therapeutic efficacy of chemotherapeutic agents by enhancing drug pharmacokinetics, reducing systemic toxicity, and prolonging circulation time (218). Peng et al. designed a co-delivery system using thermosensitive magnetic cationic liposomes (TSMCLs) loaded with Doxorubicin (DOX) and SATB1 shRNA. This system employs a magnetic field to target tumors and releases DOX in a heat-triggered manner. The combination of DOX and SATB1 shRNA resulted in enhanced inhibition of gastric cancer growth both *in vitro* and *in vivo* compared with individual treatments (184). This system offers significant potential for combining chemotherapy with gene therapy in treating gastric cancer.

Immunoliposomes Targeting Cancer-Initiating Cells (CICs): Antibody-targeted nanoparticles have been explored for improving drug delivery in cancer therapy, especially for enhancing the therapeutic effects of chemotherapy by delivering drugs directly to cancer cells (188). Yang et al. developed immunoliposomes to deliver SATB1-siRNA specifically targeting CICs in gastric cancer. CICs are highly aggressive and contribute to tumor recurrence and metastasis. Silencing SATB1 in these cells has reduced their proliferation by approximately 80% and decreased their population by 60% *in vitro* (185). Although these results are promising, further validation in animal models is required to confirm the therapeutic potential of this approach.

Gene therapy approaches targeting SATB1 are rapidly advancing, with both viral and non-viral vectors showing promising results in preclinical cancer models. By combining gene silencing with chemotherapy and utilizing advanced delivery systems such as nanoparticles and immunoliposomes, SATB1-targeted therapies can offer a novel strategy to combat aggressive cancer types. Despite these advances, further research is required to optimize these methods, evaluate their long-term safety, and confirm their clinical efficacy in treating cancers.

## 7 Discussion

SATB1 is a key genome organizer that plays a crucial role in regulating gene expression by restructuring chromatin architecture, significantly influencing cancer progression. SATB1 has been implicated in the development, invasion, and metastasis of several cancer types, including breast cancer (47), CRC (150), gastric cancer (117, 151, 152), pancreatic cancer (123, 124), OC (24, 29), endometrial cancer (97), and cervical cancer (142). SATB1 expression is frequently associated with poor tumor differentiation, aggressive phenotypes, and reduced patient survival, making it a valuable marker for poor prognosis in these malignancies. Research has shown that SATB1 promotes cancer progression primarily by inducing EMT by regulating EMT-related proteins (23, 25, 26, 32, 33). In cancers such as breast cancer and CRC, SATB1’s role in activating the Wnt/β-catenin signaling pathway has been associated with metastasis (22, 35). Loss-of-function studies further validated the critical role of SATB1 in maintaining the invasive and proliferative characteristics of cancer cells (23, 38, 90, 100). Interestingly, SATB1’s role appears to be context-dependent. In lung cancer, higher SATB1 expression has been correlated with improved patient outcomes (107, 108). Similarly, in clear cell renal cell carcinoma, elevated SATB1 levels are associated with better OS, a relationship potentially regulated by miR-21-5p, which modulates SATB1 expression (76). These findings underscore the complexity of SATB1 regulation across different cancer types.

Given its consistent role across multiple tumor types, SATB1 shows promise as a molecular marker for both diagnostic and prognostic testing in oncology. Several chemical agents, such as baicalein, hydrophobic statins, ganodermanontriol, and nicotinamide, have been identified for their ability to modulate SATB1 expression, leading to decreased tumor cell proliferation and metastasis (22, 136, 181, 182). Additionally, SATB1 silencing using RNA interference techniques, including siRNA and shRNA, has demonstrated the ability to inhibit tumor cell proliferation and invasion in preclinical models (23, 26, 33, 102, 103), positioning SATB1 as a promising therapeutic target. For example, Mao et al. developed a lysosomal adenovirus (ZD55) carrying SATB1 shRNA that significantly reduced prostate cancer growth and metastasis in experimental models (185). Moreover, co-delivering DOX and SATB1 shRNA using a nanoparticle system notably inhibits gastric cancer growth (184).

Despite these promising findings, the development of SATB1-targeted therapies remains in its infancy. The molecular mechanism by which SATB1 drives cancer progression has not yet been fully elucidated, and concerns over the potential toxicity and off-target effects of SATB1 inhibition need to be addressed. Consequently, further studies are required to deepen our understanding of SATB1’s role in cancer biology, refine therapeutic approaches, and evaluate their potential side effects. These investigations will be critical in determining the clinical applicability of SATB1 as a therapeutic target in cancer treatment.
